# *Notes from the Field:* Assessment of Awareness, Use, and Access Barriers to Cooling Centers in Maricopa County, Arizona — August 1–September 15, 2023

**DOI:** 10.15585/mmwr.mm7414a4

**Published:** 2025-04-24

**Authors:** Aaron Gettel, Meaghan Batchelor, Jessica Bell, Heather L. Walker, Kathryn G. Burr, June Vutrano, Angela Moreth, Jessica R. White, Rebecca Sunenshine, Ariella P. Dale, Jackie Ward, Nicole M. Jarrett

**Affiliations:** ^1^Maricopa County Department of Public Health, Phoenix, Arizona; ^2^Epidemic Intelligence Service, Division of Workforce Development, CDC; ^3^Arizona Department of Health Services; ^4^Career Epidemiology Field Officer Program, Division of State and Local Readiness, CDC.

**Summary**

**What is already known about this topic?**

Heat-related fatalities in Maricopa County, Arizona increased from 61 deaths in 2014 to 645 in 2023. During this period, the number of cooling centers doubled to 112.

**What is added by this report?**

In this cooling center evaluation involving 944 cooling center visitors and 1,260 general public respondents in Maricopa County during summer 2023, street signage was considered the best way to advertise cooling centers by 56% of visitors and 69% of general public respondents. A majority of visitors indicated they would like centers to be open until at least 7 p.m. Lack of transportation to centers was the most common barrier to use, described by 31% of visitors.

**What are the implications for public health practice?**

To increase access to cooling centers, Maricopa County will focus on increasing street signage, expanding operating hours, and reducing transportation barriers.

Heat-related deaths* in Maricopa County, Arizona (population approximately 4.5 million) increased approximately tenfold from 61 in 2014 to 645 in 2023 (*1*), and the number of cooling centers (volunteer facilities such as libraries, places of worship, and community centers that provide daytime air-conditioned space and water to members of the public)^†^ doubled from 56 (*2*) to 112 (C. Warner, Maricopa Association of Governments, personal communication, October 2023). During 2019–2023, the county experienced an annual average of 36 days with a daily high temperature ≥110°F (≥43.3°C) (*3*). Drug and alcohol use, homelessness, living alone, and increased age have been identified as risk factors for heat-related deaths in Maricopa County (*1*). Maricopa County Department of Public Health (MCDPH) conducted a survey to evaluate awareness, use of, and barriers to accessing cooling centers among cooling center visitors (visitors) and potential visitors (the public). This activity was reviewed by CDC, deemed not research, and was conducted consistent with applicable federal law and CDC policy.^§^

## Investigation and Outcomes

During August 1–September 15, 2023, MCDPH surveyed visitors and the public, using a 40-question Research Electronic Data Capture survey^¶^ (version 14.1.1; Vanderbilt University). The visitor and public surveys were conducted in English and Spanish using Internet-based and paper formats. At least one cooling center was selected from each of the five MCDPH geographic regions** in a zip code with high heat illness or deaths and higher Social Vulnerability Index (*4*) for that region.

The survey of visitors was conducted by trained MCDPH personnel and volunteers; respondents received a heat-relief kit^††^ for their participation. Outreach for the public survey included social media posts and press releases with direct links to the survey website. A community organization that serves older adults and persons of lower socioeconomic status, groups at increased risk for heat-related outcomes (*5*), administered the survey in-person to ensure inclusion of persons potentially at risk for heat-related illnesses or deaths who might not have online survey access. The study sample included 944 visitors to 15 cooling centers and 1,260 members of the public, 60% of whom completed the survey online and 40% in-person. Median per-question skip rates for the visitor and general survey were 9% (range = 2%–38%) and 4% (range = 1%–23%) respectively; missing data were excluded from the analyses at the question level. The average daily high temperature during the study period was 108°F (42.2°C) (SD = 6.2) with 21 days 110°F (43.3°C) or higher (*3*).

Compared with the public, a higher percentage of cooling center visitors reported experiencing homelessness (65% versus 12%), were persons of color^ §§^ (43% versus 32%), reported having a disability (18% versus 11%), and using nonprescription or street drugs (21% versus 4%), whereas a higher percentage of the public than cooling center visitors were aged ≥65 years (45% versus 16%) (Table). Many cooling center visitors (68%) and public respondents (61%) were aware of cooling centers before the survey (some visitors were unaware of cooling centers as a formal designation before taking the survey). Visitors were more likely than members of the public to have heard about cooling centers through word of mouth (47% versus 13%); the public were more likely than visitors to hear about the centers from television or radio announcements (36% versus 4%). Street signs were considered effective advertisement by both groups (56% visitors; 69% public). The median time visitors recommended that cooling centers stay open until was 7 p.m. (IQR = 6 p.m.–8 p.m.) Among persons who reported visited cooling centers for heat relief during the previous 30 days, 49% of visitors and 41% of the public visited five or more times. Approximately one half of visitors (54%) walked to reach the center, and 40% used public transportation; approximately three quarters of public respondents reported they would drive (76%). Common barriers to accessing cooling centers included lack of awareness (36% visitors; 49% public), uncertainty of locations (17% visitors; 22% public), and transportation challenges for visitors (31%). Visitors (10%) and the public (33%) indicated a desire to bring pets or emotional support animals to cooling centers. Unlike service animals, these animals can represent barriers because not all centers allow pets or emotional support animals.^¶¶^

**TABLE:** Maricopa County Department of Public Health assessment of awareness, use, and access barriers to cooling centers — Maricopa County, Arizona, August 1–September 15, 2023

Respondents	No. (%)*	
	Cooling center visitors	Public
**Total**	**944**	**1,260**
**Populations within Maricopa County^†;§^**		
**Total responses**	**812**	**1,219**
Immigrant	31 (4)	118 (10)
Lives alone	126 (16)	233 (19)
Person with disabilities	148 (18)	130 (11)
Refugee	11 (1)	45 (4)
**Total responses^¶^**	**788**	**1,201**
Person who uses drugs^¶^	163 (21)	54 (4)
Persons experiencing homelessness**		
**Total responses**	**944**	**1,260**
Reported experiencing homelessness	373 (40)	55 (4)
Reported having unstable residence	575 (61)	136 (11)
Either	618 (65)	148 (12)
**Race or ethnicity**		
**Total responses**	**836**	**1,153**
Alaska Native, American Indian, or Native American	62 (7)	21 (2)
Asian	15 (2)	25 (2)
Black or African American	117 (14)	49 (4)
Middle Eastern or North African	—^††^	—^††^
Native Hawaiian or Pacific Islander	—^††^	—^††^
White	351 (42)	708 (61)
Hispanic or Latino	157 (19)	272 (24)
Multiracial	115 (14)	56 (5)
I don’t know	11 (1)	10 (1)
**Age group, yrs**		
**Total responses**	**780**	**1,183**
18–34	205 (26)	170 (14)
35–49	251 (32)	220 (19)
50–64	202 (26)	257 (22)
65–74	96 (12)	331 (28)
≥75	26 (3)	203 (17)
Age, yrs; median (IQR)	45 (34–59)	62 (43–72)
**Were you aware that cooling centers exist in Maricopa County before taking this survey?**		
**Total responses**	**890**	**1,244**
Yes	605 (68)	765 (61)
No	285 (32)	479 (39)
**How did you find out about cooling centers?^†^**		
**Total responses**	**886**	**1,239**
I was unaware before taking this survey^§§^	148 (17)	403 (33)
I have known about cooling centers for a long time	59 (7)	84 (7)
I heard about them through someone I know (word of mouth)	415 (47)	161 (13)
I heard about them through the county or my city, a local organization, or a nonprofit or community-based organization	159 (18)	272 (22)
I saw an advertisement from the street (saw a sign)	102 (12)	100 (8)
Maricopa Association of Governments website/Heat Relief Network	30 (3)	64 (5)
Newspaper	21 (2)	89 (7)
Online article or social media (e.g., Facebook, Twitter, or Instagram)	27 (3)	217 (18)
Television or radio	39 (4)	448 (36)
Other	61 (7)	15 (1)
**What do you think are the best ways to notify people about the locations of cooling centers?^†^**		
**Total responses**	**894**	**1,237**
Advertisement from the street (e.g., cooling center sign)	503 (56)	853 (69)
Email	122 (14)	254 (21)
Internet or social media	349 (39)	740 (60)
Newspaper or online articles	191 (21)	388 (31)
Television or radio	266 (30)	751 (61)
Word of mouth	540 (60)	538 (43)
Other	122 (14)	105 (8)
**In the last 30 days, how often have you visited a cooling center to get away from the heat?** ^¶¶^		
**Total responses**	**923**	**116**
This is my first visit	202 (22)	NA
1–4 times	NA	24 (21)
2–4 times	218 (24)	NA
5–7 times	91 (10)	7 (6)
8–10 times	70 (8)	—^††^
≥11 times	240 (26)	—^††^
Never	102 (11)	75 (65)
**If you visited a cooling center before today, how much time do you typically spend at a cooling center to get away from the heat?**		
**Total responses**	**759**	**39**
This is my first time using a cooling center to get away from the heat	101 (13)	NA
<1 hr	111 (15)	—^††^
1–4 hrs	278 (37)	21 (54)
>4 hrs	269 (35)	—^††^
**What time of the day do you think that cooling centers should be open?**		
**Total responses**	**581**	NA***
Start time, median (IQR)	9 a.m. (7 a.m.–10 a.m.)	NA
End time, median (IQR)	7 p.m. (6 p.m.–8 p.m.)	NA
**How do you normally travel or how would you travel to a cooling center?^†,†††^**		
**Total responses**	**903**	**1,211**
Agency pickup (e.g., dial-a-ride or shuttle)	59 (7)	47 (4)
Bike	145 (16)	120 (10)
Drive myself	184 (20)	920 (76)
Friend, family member, or neighbor	62 (7)	191 (16)
Public transportation (bus or light rail)	364 (40)	209 (17)
Uber, Lyft, or taxi	39 (4)	80 (7)
Walk	492 (54)	288 (24)
Other	26 (3)	19 (2)
**Have any of the following kept you from visiting a cooling center when you wanted to?^†^**		
**Total responses** ^§§§^	**653**	**762**
Concerns about feeling welcome	42 (6)	30 (4)
Concerns about safety	72 (11)	81 (11)
Cooling centers are not open when I can access them	93 (14)	45 (6)
Do not want to be seen at a cooling center	15 (2)	9 (1)
I do not know how to find the location of a cooling center	114 (17)	170 (22)
I do not want to transport my belongings	53 (8)	17 (2)
I go somewhere else to cool off instead	50 (8)	157 (21)
Need to care for family or friends	29 (4)	26 (3)
No transportation	203 (31)	62 (8)
Wasn’t aware cooling centers exist	236 (36)	374 (49)
Pets might not be allowed	47 (7)	89 (12)
There is nowhere to store my belongings	67 (10)	28 (4)
Too crowded	90 (14)	41 (5)
Other	44 (7)	17 (2)
**Do you have any pets or service animals that you would want to bring with you to a cooling center if you visited?**		
**Total responses**	**815**	**1,227**
No	688 (84)	751 (61)
Yes	127 (16)	476 (39)
Would bring a pet or emotional support animal^†^	78 (10)	409 (33)
Would bring an Americans with Disabilities Act service animal^†^	31 (4)	45 (4)

**Abbreviation:** NA = not applicable.

* Missing values have been excluded from totals. Median per-question skip rates for the visitor and general survey were 9% (range = 2%-38%) and 4% (range = 1%–23%) respectively.

^†^ Question allowed more than one response; totals might exceed 100%.

^§^ The full list of response options for this question includes populations at higher risk and those that are underserved from the CDC Health Disparities Grant OT21–2103, as well as those listed in the Maricopa County Department of Public Health Community Health Needs Assessment. Data for additional response options (e.g. military member or veteran) are available online at https://www.maricopa.gov/DocumentCenter/View/92027/Cooling-Center-Visitor-Survey-Community-2023-Report and https://www.maricopa.gov/DocumentCenter/View/92026/Cooling-Center-General-Survey-Community-2023-Report

^¶^ A separate survey question ascertained respondents’ drug use.

** Respondents who reported experiencing homelessness or who indicated they have unstable housing were classified as persons experiencing homelessness.

^††^ Suppression rules applied for counts fewer than five to protect identity.

^§§^ Survey design allowed all respondents to answer this question and the previous question. Counts might not align across question responses because respondents might have been unaware of cooling centers as a formal designation before taking the survey, but recalled learning via word of mouth or from organizations about places where they might go to stay cool.

^¶¶^ Response options for this question differed between cooling center visitors and the public. Because public survey respondents were not surveyed at cooling centers, the response, “This is my first visit” was not relevant to public respondents and therefore not provided as an option. The first range for the public survey was one to four times.

*** The question was not posed to the public because it was replaced with another question.

^†††^ Visitors were asked how they normally travel to cooling centers; public respondents were asked how they would travel to cooling centers.

^§§§^ Respondents who indicated they do not experience barriers to visiting a cooling center (174 visitors; 207 public) were removed from the denominator to maintain focus on those who experience barriers.

## Preliminary Conclusions and Actions

The results included in this report highlight diversity of current and potential cooling center users, underscoring the need for inclusive strategies in increasing awareness and accessibility. To increase awareness and visibility of and access to cooling centers, MCDPH incorporated the following into its community heat action plan: expanding operation hours until 7 p.m. or later at 17 centers, making additional street signage available, and funding a heat-relief call center staffed by bilingual health workers to facilitate location of and transportation to and from cooling centers.

## Footnotes

* Heat-associated mortality cases were identified using *International Classification of Diseases, Tenth Revision* codes X30 (exposure to excessive natural heat), T67.X (effects of heat and light), and P81.0 (environmental hyperthermia of newborn) or by the key phrases of heat exposure, environ, exhaustion, sun, heat stress, heat stroke, and hyperthermia on part I or II of death certificates.

† In 2023, cooling centers, which included respite centers, operated during May 1–September 30, did not track visitor counts, and did not operate under standardized protocols. Hours of operation were generally 9 a.m.–5 p.m. weekdays. Respite centers later became an official heat relief site type distinguished from cooling centers. https://azmag.gov/Programs/Heat-Relief-Network/Resources

§ 45 C.F.R. part 46, 21 C.F.R. part 56; 42 U.S.C. Sect. 241(d); 5 U.S.C. Sect. 552a; 44 U.S.C. Sect. 3501 et seq.

¶ Visitor and public surveys are available as report appendices at https://www.maricopa.gov/DocumentCenter/View/92027/Cooling-Center-Visitor-Survey-Community-2023-Report and https://www.maricopa.gov/DocumentCenter/View/92026/Cooling-Center-General-Survey-Community-2023-Report

** Maricopa County is divided into five regions (Northwest, Northeast, Southwest, Southeast, and Central) for its community health assessment. For specific boundaries, visit https://maricopa.maps.arcgis.com/apps/instant/basic/index.html?appid=b86a52f0a4fe4802b503ec22b251be9d

†† Heat-relief kits included a cooling towel, hygiene-related items, sunscreen, aloe vera, and socks.

§§ A person of color for this analysis was anyone who selected their race and ethnicity as Alaska Native, American Indian or Native American, Asian, Black or African American, Hispanic or Latino, Middle Eastern or North African, or Native Hawaiian or Pacific Islander.

¶¶ By law, only Americans with Disabilities Act service animals have protected access to public places, including cooling centers.
